# Microplastics and female infertility: reproductive toxicity and underlying mechanisms

**DOI:** 10.3389/fendo.2026.1867408

**Published:** 2026-08-03

**Authors:** Shuai Ma, Min Wang, Minghui Fan, Yimei Meng, Ran Sun, Caomeihui Shen, Minjia Sheng, Yinggang Zou

**Affiliations:** 1 Department of Obstetrics and Gynecology, The Second Hospital of Jilin University, Changchun, China; 2 The Reproductive Medical Center, China-Japan Union Hospital of Jilin University, Changchun, China; 3 College of Animal Science, Jilin University, Changchun, China

**Keywords:** endometriosis, environmental exposure, infertility, microplastics, polycystic ovary syndrome, premature ovarian insufficiency, reproductive toxicity

## Abstract

In modern society, the progression of ovarian aging in women has accelerated, and the incidence of infertility has increased markedly with a distinct younger-onset trend. Exploring the etiology and pathogenesis of infertility has become a core research focus for clinicians. Plastic products bring tremendous convenience to modern life, while the health risks caused by plastic pollution have aroused global concern. As typical endocrine-disrupting chemicals, microplastics (MPs) can enter the human body through dietary intake, respiration, and skin contact, and accumulate in multiple organs. Accumulated MPs impair the physiological function of the female reproductive system and are closely associated with multiple gynecological disorders, including polycystic ovary syndrome, endometriosis, and premature ovarian insufficiency. After internalization, MPs trigger a series of pathological events such as epigenetic alterations, hormonal metabolism disturbance, inflammatory response, immune dysregulation, oxidative stress, and cellular damage, ultimately resulting in female reproductive impairment. The exposure routes and pathogenic mechanisms underlying MPs-induced reproductive toxicity have emerged as critical scientific issues that urgently require addressing. From an innovative perspective, this review elaborates on the mechanisms underlying the occurrence and progression of female infertility-related diseases mediated by MPs. It aims to provide novel insights for the clinical diagnosis and treatment of female infertility, offer theoretical references for pre-pregnancy counseling and reproductive health management, and supply scientific evidence for the precise prevention and control of MP pollution.

## 1 Introduction

With the development of the social economy, the transformation of human lifestyles, and the worsening of environmental pollution, the issue of declining human fertility has become increasingly prominent. Delayed childbearing age has evolved into a global concern, and the number of infertile patients worldwide continues to rise year by year (1). The World Health Organization has predicted that infertility will become the third most common human disease in the 21st century, second only to tumors and cardiovascular diseases (2). Reduced human fertility is not merely a reproductive health concern but also triggers a series of social, economic, and family-related problems (3). Multiple contributing factors are responsible for female infertility, and the primary female-induced causes mainly include ovulation disorders, tubal lesions, endometrial abnormalities, and immune dysregulation (4). Modern human life is highly dependent on plastic products (5).

With inherent merits such as low weight, high durability, favorable ductility, and excellent portability, plastics dominate the production of daily consumer goods (6). Plastic components pervade nearly all commercial products, particularly packaging materials, cosmetics, household items, electronic devices, and other daily necessities. When plastic products are used and disposed of, and undergo physical abrasion, chemical degradation, and other weathering processes, they continuously generate microplastics (MPs) (7). The concept of “MPs” was first proposed by Thompson et al. in 2004, after which MPs attracted widespread public attention (8). Restricted waste recycling systems and inadequate regulations on plastic waste management, combined with the non-degradable nature of MPs, pose severe challenges to the global environment. Beyond ecological damage, MPs can accumulate in organisms and be transmitted through the food chain, ultimately posing severe threats to human health (9). Current studies have identified MPs in a broad range of human tissues and organs, including the gastrointestinal tract, liver, lung, kidney, testis, uterus, and ovary (10). *In vitro* studies have demonstrated that 100 μg/mL polystyrene (PS) can inhibit the proliferation of ovarian granulosa cells (OGCs), induce cell apoptosis, and trigger excessive accumulation of reactive oxygen species (ROS). *In vivo* animal experiments further verified that continuous exposure to 1 mg/day PS impairs fertility, disrupts ovarian function, and aggravates ovarian apoptosis in female mice (11). Therefore, in-depth exploration of the interaction mechanisms between MPs and infertility-related diseases is conducive to providing novel insights for the diagnosis and clinical treatment of female infertility.

## 2 Infertility

Infertility has long remained a major challenge in reproductive medicine (12). The major underlying reproductive disorders that lead to female infertility mainly include polycystic ovary syndrome (PCOS), endometriosis, and premature ovarian insufficiency (POI). PCOS is one of the most common reproductive endocrine disorders among women of childbearing age and is a leading cause of ovulatory infertility during the reproductive period (13). Current research still cannot fully clarify its etiology and pathogenesis. Clinically, PCOS is characterized by irregular menstruation, hyperandrogenic manifestations or hyperandrogenemia, and polycystic ovarian morphology under ultrasonography, with infertility as a prominent clinical manifestation. Infertility is a prominent clinical manifestation of this disorder (14). MPs exacerbate the PCOS phenotype through various mechanisms: they directly trigger excessive androgen synthesis in ovarian theca cells and induce insulin resistance to amplify endocrine disturbances, thereby worsening ovulatory failure and PCOS-related infertility.

Endometriosis is a common gynecological disease with a gradually increasing incidence (15). Pain and infertility caused by endometriosis seriously impair women’s physical and mental health (16). The retrograde menstruation is the most widely accepted pathogenic theory of endometriosis: endometrial tissues flow backward into the peritoneal cavity during menstruation. Accumulated MPs in endometrial tissues may enhance the adhesiveness and survival capacity of ectopic endometrium after retrograde flow, which exacerbates the development and progression of endometriosis. For patients with endometriosis, the natural pregnancy rate declines progressively with the extension of postoperative time. Moreover, poor oocyte and embryo quality in endometriosis patients inevitably leads to a low implantation rate during assisted reproductive technology treatment (17).

POI is a multifactorial reproductive endocrine disease. Multiple pathogenic factors, including genetic abnormalities, immune disorders, environmental pollutants, oxidative stress, and chronic inflammation, are closely involved in the occurrence of POI (18). Researchers have yet to uncover the pathogenesis of POI fully. Beyond reproductive dysfunction, POI also elevates the risks of depression, anxiety, cognitive decline, premature death, osteoporosis, cardiovascular diseases, and other comorbidities (19). Infertile couples generally seek fertility treatment via assisted reproductive technology; however, the complicated procedures, prolonged treatment cycles, and high medical costs further impose severe psychological and economic burdens on affected patients (20).

The potential adverse effects of MP exposure on the reproductive health of women of childbearing age have drawn increasing public attention. MPs accumulate in female reproductive organs such as the ovaries and uterus, thereby interfering with ovulation, fertilization, and embryo implantation (21). Environmental MPs are absorbed through daily intake and accumulate in reproductive tissues, eventually triggering functional abnormalities in the ovary and uterus (22). Zurub et al. demonstrated that long-term ovarian damage induced by nanoplastics (NPs) exposure is closely associated with aberrant methylation of core reproductive genes, which directly results in oocyte developmental arrest (23). There is an urgent need to further explore the molecular mechanisms underlying MP’s involvement in the progression of female infertility-related diseases, as this has important clinical implications for improving female fertility.

## 3 MPs

### 3.1 Classification and distribution of MPs

As of 2015, 630 million metric tons of plastic waste had been generated; only 9% was recycled, while 79% accumulated in the environment. If current trends continue, this figure is projected to reach 1.2 billion metric tons by 2050 (24). Researchers define MPs as insoluble solid plastic particles smaller than 5 mm in diameter, which mainly stem from plastic degradation and industrial fragmentation (25). According to different sources, MPs fall into two categories: primary and secondary MPs. Primary MPs mainly include microbeads in personal care products and synthetic fibers shed from textiles, while secondary MPs refer to fragments formed by the aging and degradation of large plastic debris, as well as fibers released during fabric washing (26).

Morphologically, MPs present diverse forms, including spheres, granules, fragments, fibers, and irregular particles. MP pollution now prevails worldwide (27). MPs are ubiquitously detected in daily foods, including fruits, vegetables, aquatic products, meat, dairy products, and condiments such as salt, sugar, and honey (28, 29). Meanwhile, over 20 types of polymers exist in the human body. Among them, polyethylene terephthalate (PET), PS, polypropylene (PP), polyvinyl chloride (PVC), polycarbonate (PC), and polyethylene (PE) are the most predominant types. Mechanical friction and thermal stimulation enable PP and PE plastics to release large quantities of MPs into food matrices (30). Accumulating evidence has confirmed the presence of MPs with diverse compositions, morphologies, and abundance in multiple human organs, body fluids, and excreta (31) (**Table 1**). Leslie et al. verified the existence of MPs in human blood and hypothesized that MPs could regulate the expression of inflammation−related miRNAs, such as miR−146a and miR−21 (32).

**Table 1:** Distribution characteristics of MPs in the human body.

Body parts	MPs level	Particle size	Polymer type	Assay method	Reference
Blood	1.6μg/mL	≥700nm	PET, PE	Pyrolysis gas chromatography-mass spectrometry (PyGC/MS)	(32)
Placenta	3	<10μm	PP	Raman	(85)
Thrombus	1	2.1-26.0μm	LDPE	Raman	(70)
Uterus	(1.5 ± 1.2)/g	2-200μm	PE, PP	Raman	(75)
Heart	–	20-469μm	PET, PU, PA	Laser direct infrared (LDIR)	(130)
Brain	4026-5608μg/g	<200nm	PET, PU, PA	PyGC/MS	(131)
Urine	7	4.0-15.0μm	PP, PA, PS	Raman	(132)
Breast milk	58	2.0-12.0μm	PP, PE, PVC	Raman	(133)
Liver	0.7/g	3.0-29.5μm	PET, PS, PVC	Raman	(134)
Kidney	0.5/g	3.0-29.5μm	PET, PS, PVC	Raman	(134)
Spleen	1.1/g	3.0-29.5μm	PET, PS, PVC	Raman	(134)
Intestine	(28.1 ± 15.4)/g	0.8-1.6mm	PC, PA, PP	Fourier transform infrared (FTIR)	(135)
Testis	(11.60 ± 15.52)/g	21.76-286.71μm	PVC, PE, PS	PyGC/MS	(136)
Blood	1.84-4.65μg/mL	5-3000μm	PE, PA	FTIR	(137)
Meconium	54.1/g	20-500μm	PU, PA	LDIR	(138)
Placenta	18/g	20-500μm	PU, PA	LDIR	(138)
Placenta	(2.70 ± 2.65)/g	>20μm	PVC, PP	LDIR	(139)
Placenta	(126.8 ± 147.5)μg/g	5-10μm	PP	Raman	(140)

**Figure 1:**
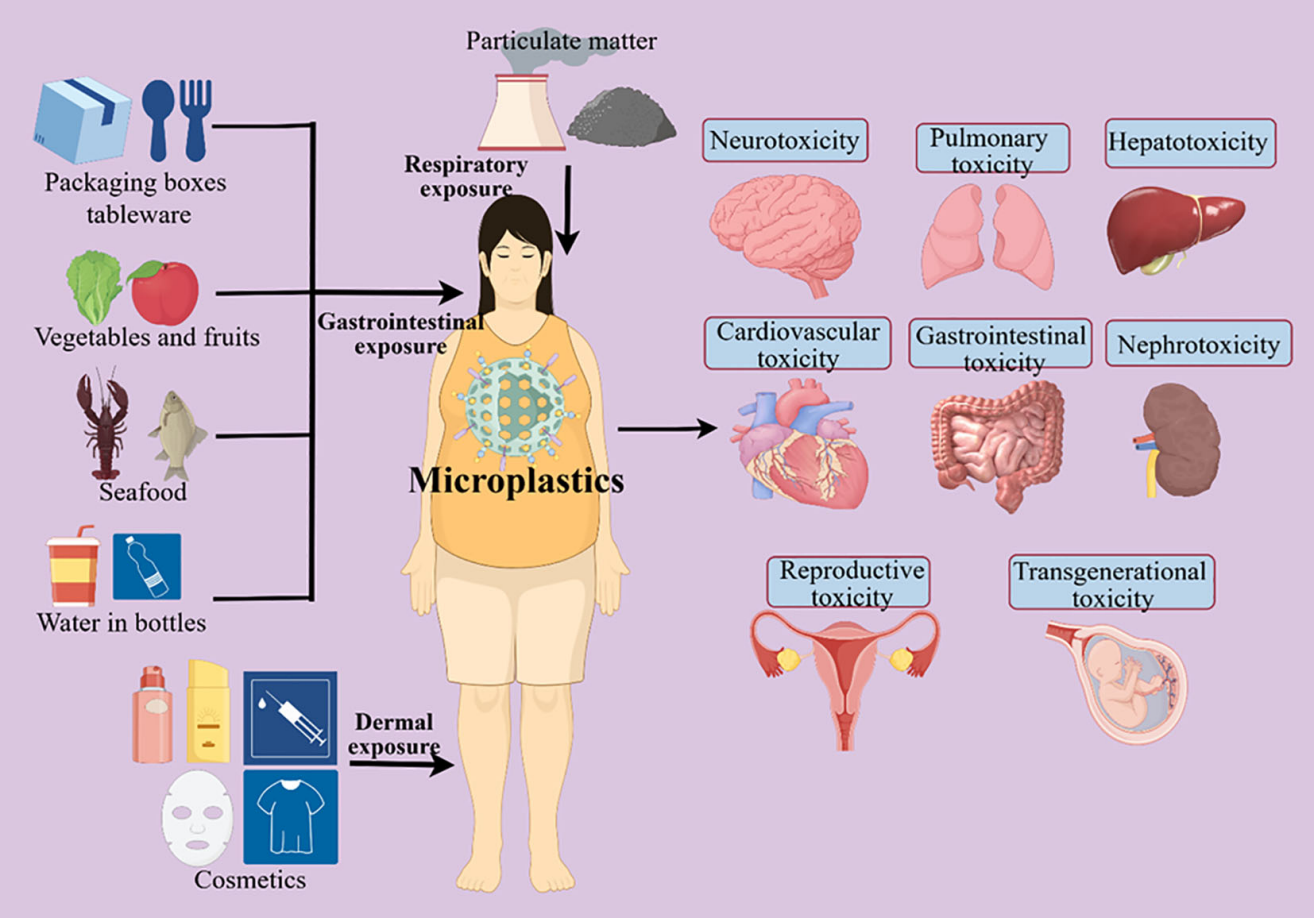
Exposure sources of MPs and potential risks to human health. The exposure pathways for MPs include gastrointestinal, respiratory, and dermal exposure. MPs accumulate in various organs throughout the body and pose potential risks to human health.

Studies confirm that disposable paper cups can release up to 25,000 MPs within 15 minutes of hot water infusion (33). Hernandez et al. reported that a single plastic tea bag released approximately 11.6 billion MPs and 3.1 billion NPs into tea under specific brewing conditions (95 °C, 5 minutes) (34). However, newer research indicates that plastic particle release is highly dependent on water chemistry, temperature, and bag material (35). MPs feature tiny particle size, environmental persistence, bioaccumulation risks, latent hazardous effects, and powerful adsorption capacity (36). They readily adsorb various hazardous substances, including arsenic, per− and polyfluoroalkyl substances, phthalic acid esters, polycyclic aromatic hydrocarbons, and bisphenol A, all of which are typical endocrine−disrupting chemicals (37–39). Previous studies have confirmed that the combined exposure to MPs and phthalates induces stronger reproductive toxicity than single exposure to either contaminant alone (40). MPs disrupt the synthesis, transport, metabolism, and binding of endogenous hormones, leading to neurotoxicity. More importantly, such hormonal disorders will disturb the normal function of the female reproductive endocrine system. Combined with adsorbed pesticides and other pollutants, MPs produce synergistic toxicity, damage biological barriers, and affect immune function. As ubiquitous pollutants, MPs can accumulate in the human intestine, blood, and placenta (41) (**Figure 1**). These tissues act as important transport and carrier pathways, enabling MPs and associated contaminants to invade female reproductive organs, trigger tissue damage, and eventually contribute to female infertility (42).

### 3.2 Exposure pathways of MPs

#### 3.2.1 Gastrointestinal exposure

Dietary intake represents the primary route for MPs to enter the human body, and the digestive system is one of the earliest systems exposed to environmental MPs (43). MPs contained in food pass through the oral cavity and subsequently enter the pharynx, esophagus, stomach, intestine, and other digestive organs. Intestinal epithelial cells primarily mediate MP absorption via endocytic transport, while M cell phagocytosis and receptor−dependent endocytosis also contribute to this process (44). Ingested MPs can disrupt gastric juice secretion, compromise gastric barrier function, and inhibit gastric epithelial cell proliferation (45). In addition to gastrointestinal impairment, ingested MPs also induce systemic exposure through body fluid circulation, thereby triggering immune injury and oxidative stress (46). MPs smaller than 150 μm translocate across the mammalian intestinal tract into lymphatic and circulatory systems, inducing systemic exposure, and eventually exit the body through feces, urine, and other routes (47). After entering the circulation, MPs adhere to erythrocyte membranes, induce erythrocyte aggregation and vascular endothelial adhesion, and may even lead to hemolysis in severe cases (48). Furthermore, circulating MPs can migrate to the liver, reproductive system, and brain, thereby triggering hepatotoxicity, reproductive toxicity, and neurotoxicity, respectively (49, 50).

#### 3.2.2 Respiratory exposure

The respiratory system serves as another critical route for MPs to enter the human body. Large-sized MPs mainly settle in the upper respiratory tract, such as the nasal cavity and pharynx. In contrast, MPs smaller than 5 μm, particularly NPs with diameters below 100 nm, can penetrate deeply into the respiratory tract, accumulate in the lungs, and even cross the alveolar barrier to enter the systemic circulation (51). Animal experiments in mice with intratracheal instillation demonstrated that the lung exhibits the most prominent accumulation of MPs, followed by the heart, liver, and intestine (52). For adults and minors exposed to ambient air for 2 hours daily, their total annual exposure to atmospheric MP (inhalation, dust ingestion, and dermal contact) reaches 1.06×10^5^ particles and 7.37×10^4^ particles (53). The multi-pathway exposure to atmospheric MPs may cause reproductive impairment and elevate the risk of female infertility. Populations with occupational exposure to MP are at a higher risk of developing asthma, alveolitis, chronic bronchitis, and chronic pneumonia (54). *In vivo* studies have revealed that continuous MP inhalation reduces the inspiratory duration, respiratory rate, and expiratory time in rats. The expression levels of pulmonary fibrosis-related factors are positively correlated with MP exposure concentration, ultimately leading to the occurrence of pulmonary fibrosis in mice (55).

#### 3.2.3 Dermal exposure

People commonly find NPs in daily personal care commodities, such as facial cleansers, body wash, and cosmetics (56). Researchers define NPs as plastic particles with diameters less than 100 nm. Specifically, 20 nm PS particles primarily accumulate in skin hair follicles and hardly penetrate the stratum corneum, while PS particles ranging from 20 to 200 nm only infiltrate the outermost layer of the skin (57, 58). Ultraviolet radiation can induce skin damage and subsequently enhance the percutaneous absorption of NPs (59). Medical invasive procedures, such as intravenous infusion and injection, serve as an emerging exposure route, since MPs released from medical supplies directly contact human blood and tissues (60). Based on an exposure assessment model established by Li et al., the annual intake of MPs per individual via syringes, infusion sets, and indwelling venous needles is estimated to range from 0.35 to 6.22 particles (61).

#### 3.2.4 Bioaccumulation

MPs can enter the organism and accumulate in multiple organs. The particle size of MPs determines their absorption efficiency. MPs smaller than 10 μm are absorbed through the gastrointestinal tract into the circulatory system and accumulate in the liver, kidneys, and brain. MPs less than 5 μm tend to deposit in macrophages, lymph nodes, the blood circulation, and the spleen. Particles below 0.1 μm are capable of penetrating cell membranes and the placental barrier. Mohamed et al. reported that the irreversible accumulation of MPs could reach 8.32×10^3^ and 5.01×10^4^ particles in humans by the age of 18 (children) and 70 (adults) (22). The abundance of MPs in sea salt is markedly higher than that in lake salt and rock salt (62). Assuming a daily salt intake of 5 g per capita, the annual cumulative MP intake solely from sea salt reaches 216 particles per person (63). Retained internal MPs can cross biological barriers and invade distant organs. Wang et al. detected PE and PVC in renal transplant donor tissues, with concentrations ranging from 25.7 to 98.9 μg/kg and 31.3 to 65.4 μg/kg, respectively (64). Mounting evidence highlights the detrimental effects of MP exposure on the reproductive system (65, 66). In a mouse model exposed to PS-MPs, Wei et al. observed a significant increase in the proportions of macrophages and neutrophils in endometrial tissues, accompanied by an imbalance of the uterine microenvironment. Notably, these toxicological effects display intergenerational traits: MP toxicity can pass to offspring and further heighten systemic health risks (67). As demonstrated by Martins et al., continuous exposure to MP caused a 10% mortality rate in the F0 generation and impaired female reproductive capacity. When F0 female daphnids were transferred to clean media without MPs, their offspring quantity partially recovered; nevertheless, low reproduction efficiency and population growth rates persisted until the F3 generation. In contrast, sustained exposure to MP resulted in completely immobile juveniles in the F1 generation, leading to the failure of subsequent population renewal (68).

### 3.3 Health risks and toxic effects

MPs pose significant health risks to humans. They act as a high-risk factor for cardiovascular diseases. Individuals carrying MPs within arterial plaques show 3.53-fold greater risks of heart disease, stroke, and mortality (69). MP abundance in thrombi correlates positively with platelet counts, and MPs trigger thrombosis by facilitating platelet activation (70). The detection of MPs in human bone marrow also serves as a critical risk factor for the development of hematological malignancies (71). MPs penetrate the blood–brain barrier, enter immune cells via phagocytosis, and accumulate to obstruct cerebral microvessels in mice. NPs can invade brain tissues and interact with α-synuclein in neurons, thereby facilitating the formation and propagation of pathological protein fibrils, triggering lysosomal damage, and ultimately elevating the risk of Parkinson’s disease (72). Mounting studies uncover MPs across multiple tissue samples of the female reproductive system. Researchers also identify MPs in the blood, tumor tissues, and paracancerous tissues of patients with cervical cancer (73). Exposure to MP alters the thickness of cervical glandular epithelial cells and significantly upregulates the expression of genes associated with endometriosis and other endometrial disorders (74). Xu et al. conducted qualitative and quantitative analyses of PS-MPs in human uterine leiomyoma and normal myometrial tissues. The results demonstrated that 16 tissue samples contained PS−MPs at an average abundance of 1.5 ± 1.17 particles per gram, and PS−MP concentrations correlated closely with uterine leiomyoma size (75). MPs can penetrate the endometrium and induce reproductive inflammation and fertility impairment. Notably, menstrual cycle-associated physiological dynamics play a key role in MP absorption: periodic changes in endometrial vascularity and cervical mucus viscosity alter the barrier properties of reproductive tissues, thereby regulating the penetration efficiency of MPs. Moreover, accumulated MPs could further modify the physicochemical properties of cervical mucus, including its pH, chemical composition, and viscoelasticity. Such alterations may create a hostile physical and chemical barrier that inhibits sperm survival and migration, although direct evidence in humans remains limited and warrants further investigation.

The toxic effects of MPs are not limited to local tissues; severe exposure can lead to metabolic disorders, immune system damage, and systemic pathological alterations. The primary toxic mechanisms of MPs are summarized as follows: (1) Disrupting the integrity of biological barriers, including the intestinal barrier, blood–brain barrier, cardiovascular barrier, and blood–testis barrier, which increases the susceptibility to mucosal infection; (2) Interacting with biomacromolecules: binding to hemoglobin to cause vascular obstruction, and triggering abnormal interactions with α-synuclein to aggravate the progression of Parkinson’s disease (76); (3) Inducing oxidative stress, which is linked to endoplasmic reticulum stress and mitochondrial dysfunction (77);(4) Disturbing extracellular matrix homeostasis and disrupting intracellular signal transduction pathways (78).

## 4 Mechanisms linking MPs to infertility

### 4.1 Morphological and functional damage to female reproductive tissues

**Figure 2:**
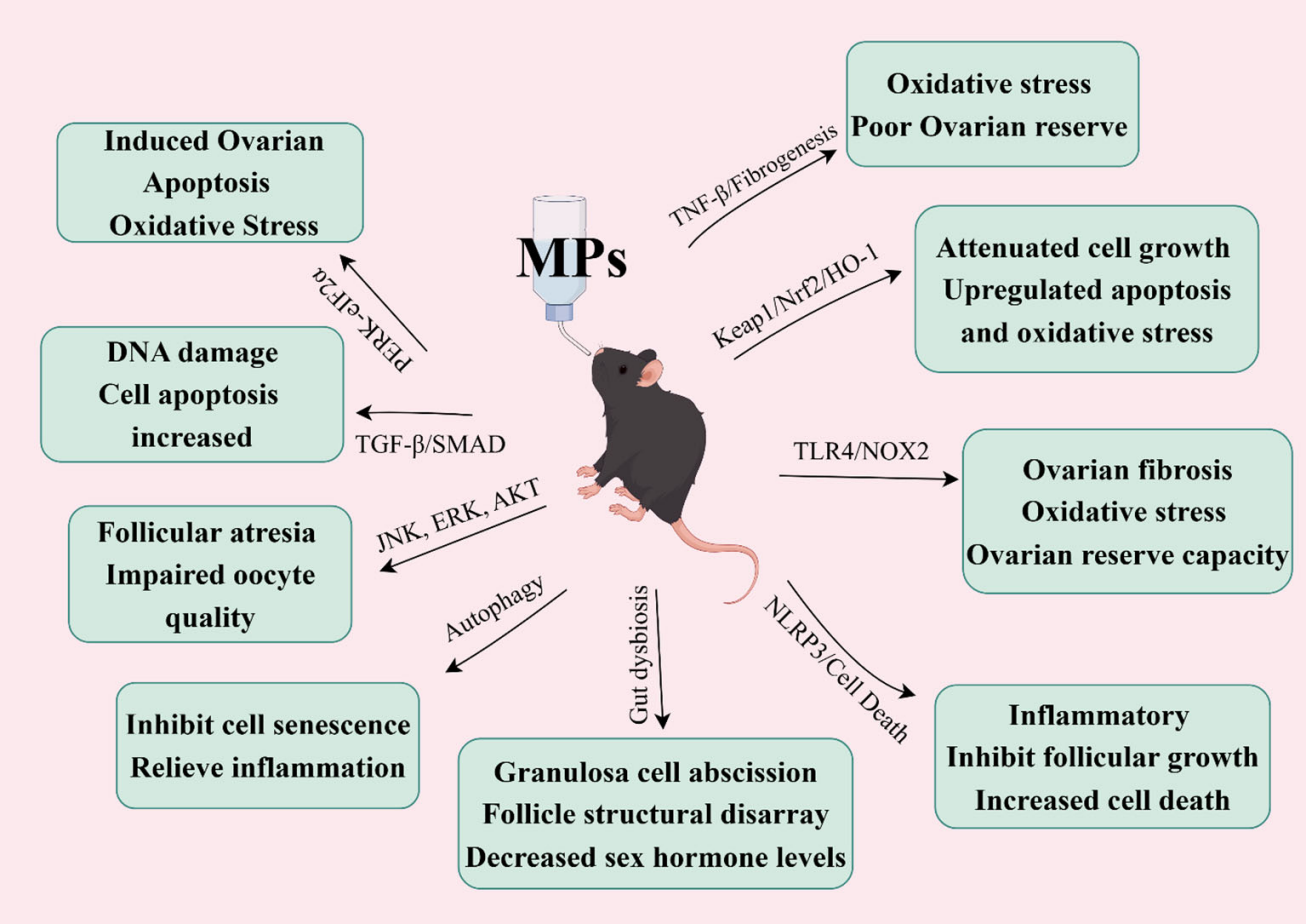
Toxic effects of MPs on the female reproductive system. MPs induce impaired ovarian and uterine function, leading to female reproductive dysfunction.

Exposure to MP induces multiple types of female reproductive toxicity, mainly manifested as structural and functional damage to the ovary, uterus, placenta, and reproductive tract, as well as adverse impacts on embryonic development and offspring health (**Figure 2**). As the core reproductive and endocrine organ, ovarian dysfunction directly leads to female reproductive disorders. Hou et al. demonstrated that MP exposure reduced the number of growing follicles and AMH in female rats. Mechanistically, MPs damage the primordial follicle pool by triggering apoptosis and pyroptosis of OGCs, which is a key pathological driver of POI and female infertility (79). Although the basic follicle recruitment and ovulation can still be maintained in MP-exposed mice during the estrous cycle, follicular development is severely disturbed, with decreased antral follicles, oocyte survival rate, and first polar body extrusion rate (80). Beyond direct follicle damage, MPs may impair ovarian function by disrupting the stromal microvasculature. Circulating MPs can physically occlude the dense ovarian capillary network and cause endothelial injury, leading to localized tissue hypoxia.

Exposure to MP impairs oocyte maturation, fertilization capacity, and embryonic development after superovulation. This toxicity is attributed to MP-induced disruption of the oocyte meiotic spindle, leading to errors in chromosome segregation and aneuploidy, which further compromise female fertility. Additionally, maternal exposure to PS-MP during gestation and lactation reduces offspring body weight and survival, and also inhibits oocyte maturation, fertilization, and embryonic development in the next generation (81). Existing comparative studies show that PS-MPs accumulate more abundantly and trigger stronger oxidative stress in ovarian tissues than in testicular tissues of mice. Female mice exhibit reduced ovarian size and follicle numbers, while male mice present decreased viable spermatogenic cells and increased sperm malformation. It should be noted that the original study did not correct for inherent differences in blood flow, tissue mass, and baseline antioxidant levels between the two organs (67). The inhibitory effects of MPs on follicle growth and maturation vary with exposure duration and dosage.

The uterus is essential for embryo implantation and successful pregnancy, and MP exposure poses a serious threat to uterine function. MP treatment reduces uterine weight and causes extensive histological lesions in the myometrium, endometrium, and uterine epithelial cells. MPs also disrupt the endometrial microenvironment, suppress endometrial stromal cell differentiation and decidualization, and impair endometrial vascular remodeling and embryonic nutrient supply, thereby increasing the risk of early miscarriage. Furthermore, MP disrupts the balance of endometrial cell proliferation and apoptosis, disturbs uterine immune homeostasis, and alters the sensitivity of estrogen and progesterone receptors via peripheral tissue accumulation (82). Wu et al. reported that PS-MPs cause severe uterine histopathological changes, including endometrial adhesion, uterine cavity stenosis, endometrial thinning, and reduced gland quantity, eventually leading to uterine fibrosis (83). Qin et al. confirmed that MPs infiltrate the endometrium in a size-dependent manner. MPs enter the uterus via the dietary-blood circulation pathway or the vaginal-uterine route; meanwhile, MPs reduce the dominance of Lactobacillus in the vagina, induce genital dysbiosis, and elevate the risk of pelvic inflammatory disease, a major cause of tubal infertility (84).

MPs also cause ultrastructural damage to syncytiotrophoblast organelles. Using Raman spectroscopy, Ragusa et al. successfully detected MPs in human placenta tissues, and a total of 12 spherical or irregular MPs were isolated from four placental samples (85). Maternal NP exposure enables particles to accumulate in placental and fetal tissues, damage endothelial trophoblasts, and disrupt uteroplacental blood perfusion, resulting in decreased fetal and placental weights (86). In pregnant women, exposure to MP leads to gut dysbiosis and systemic inflammation, disturbs glucose metabolism, and increases the risk of gestational diabetes mellitus. Combined placental injury and maternal metabolic disorders aggravate reproductive risks. Yang et al. found reduced total antioxidant capacity, downregulated claudin-3, and increased caspase−3 expression in MP-exposed mouse placentas. PS-NPs destroy placental barrier integrity, trigger excessive ROS production and cell apoptosis, and impair normal placental function (87). In zebrafish, leachates from boiling plastic bags decrease spawning capacity, embryo hatching rate, and sperm quality, and increase larval malformation. MPs also disrupt embryonic metabolic homeostasis and alter the metabolism of lysine, glucose, cholesterol, and phenylalanine (88).

### 4.2 Underlying molecular and pathological mechanisms

#### 4.2.1 Epigenetic alterations

MPs can carry and release heavy metals to induce epigenetic modifications. They cause aberrant hypermethylation or hypomethylation at CpG sites and disrupt normal gene expression (65). Stress signals triggered by MP exposure reshape histone modification patterns, potentially leading to persistent intergenerational epigenetic effects (89). PS−MP exposure dysregulates the expression of key miRNAs involved in apoptotic signaling and oxidative stress pathways. Accordingly, MPs act as key epigenetic regulators to modulate cellular homeostasis and disease susceptibility (90). Chatterjee et al. illustrated that NPs exposure impairs ovarian function, markedly downregulates genes related to oocyte maturation, and suppresses follicular development and ovulation, indicating that MPs directly interfere with gene transcription in the reproductive system (91). MPs activate the CNR1/CRBN/YY1/CYP2E1 signaling axis, which promotes excessive ROS production and provokes severe oxidative stress. Consequently, OGCs suffer from oxidative DNA damage, ultimately resulting in follicle rupture and atresia (92). Zebrafish experiments also verified that high-dose PS-MPs reduce OGC viability and increase DNA damage levels (93).

#### 4.2.2 Hormone disorders

The hypothalamus−pituitary−ovarian axis (HPOA) is a core endocrine pathway that governs female reproductive function. MPs disrupt HPOA homeostasis, leading to decreased estradiol secretion and delayed gonadal development. Haddadi et al. found that MP exposure significantly reduced estrogen levels in rats, accompanied by increased atretic follicles, OGCs degeneration, shortened estrous cycle duration, and decreased ovarian index (80). Moreover, exposure to MP significantly reduced levels of testosterone, follicle-stimulating hormone, and luteinizing hormone (94). PS−MPs interfere with the hypothalamic−pituitary−gonadal axis and disrupt steroid synthesis pathways, thereby inducing disorders of the reproductive endocrine system. In female medaka, MPs inhibit the transcription of gonadotropin-releasing hormone, follicle−stimulating hormone, luteinizing hormone, and their receptors, suppress estrogen synthesis, and hinder gonadal maturation (95).

#### 4.2.3 Multiple forms of cell death

The death of OGCs is a key driver of impaired follicular development under MP stress. MP disrupts cell membrane integrity and induces various types of cell death. A study by Xue et al. showed that PS MP downregulates the anti-apoptotic Bcl-2 and upregulates the pro-apoptotic Bax, leading to excessive apoptosis of OGCs and reproductive dysfunction (96). PS-MP activates the Wnt/β-catenin signaling pathway, promotes apoptosis in OGCs, accelerates ovarian fibrosis, and reduces ovarian reserve in rats (97). By activating the NLRP3/Caspase−1 signaling cascade, MPs induce pyroptosis and apoptosis in OGCs, disturb oogenesis, and cause progressive declines in ovarian reserve following long−term low−dose exposure, thereby accelerating reproductive aging (79). In addition, PS-NPs trigger ferroptosis in porcine oocytes, accompanied by elevated intracellular Fe^2+^ and lipid peroxidation. Ferrostatin−1 can reverse such damage (98). Ali et al. reported consistent results, noting prominent iron overload and excessive lipid peroxidation following MP exposure (99). Transcriptome and metabolome data reveal that MP reproductive toxicity is closely linked to peroxisome proliferator-activated receptor and lipid metabolism pathways (100).

#### 4.2.4 Inflammatory responses

Chronic low-grade inflammation features continuous pro-inflammatory cytokine release and abnormal expression of inflammatory markers, such as IL-1β, IL-6, TNF-α, and NF-κB (101). This persistent pathological condition strongly correlates with multiple female reproductive disorders, including PCOS and endometriosis. MPs trigger systemic inflammation, disrupt HPOA function, and ovarian hormone secretion. Saeed et al. demonstrated that chronic exposure to PS-MP upregulated IL-6 and NF-κB expression, thereby inducing chronic inflammatory responses in female rats (102). NP exposure increases pro-inflammatory cytokines in ovarian tissues and impairs follicle and corpus luteum development (91). Exposure to PE-MP induces massive inflammatory cell infiltration in the ovary and activates the TRAF-6/NF-κB signaling pathway (103). Moreover, MPs act as carriers for bisphenol A, polychlorinated biphenyls, and other pollutants, which further amplify inflammatory responses.

#### 4.2.5 Immune dysregulation

MP alters the expression of immune-related genes (104). Different doses of PE-MPs exert distinct effects on litter size and offspring sex ratio. High-dose PE-MPs reduce the proportion of T cells and inhibit dendritic cell maturation in female offspring (105). PS-MPs induce intestinal immune imbalance and increase pro-inflammatory cytokines (106), and inhibit the activity of detoxification enzymes in multiple tissues, ultimately impairing host immune function (107, 108). Veneman et al. observed that PS particles partially colocalize with neutrophils and macrophages, participate in immune recognition, and activate the alternative complement pathway, thereby triggering systemic immune responses in zebrafish (109). In mammals, including humans, MP-induced tissue injury may release cryptic ovarian autoantigens, which can trigger autoimmune oophoritis. This autoimmune disorder severely impairs ovarian function, leads to premature ovarian failure, and acts as an important pathogenic factor for female infertility.

#### 4.2.6 Oxidative stress

Excessive ROS accumulation and impaired antioxidant defense (oxidative stress) are core mechanisms of MP reproductive toxicity. MPs induce ROS overproduction in oocytes and embryos, causing mitochondrial dysfunction, cell apoptosis, ovarian inflammation, and poor gamete quality (110, 111). Huang et al. demonstrated that orally administered PS-NPs accumulate in mouse ovarian tissues, trigger oxidative stress in ovarian cells, and ultimately reduce reproductive capacity and litter size (112). Plastic leachates also increase ROS and oxidative damage markers in zebrafish (113). The expression of genes involved in oxidative stress-related signaling cascades, including the TNF-α, IFN-β, insulin, and Wnt pathways, is also dysregulated under MP toxicity (114). Exaggerated oxidative stress induces protein and nucleic acid damage, causes irreversible DNA repair failure, and deteriorates oocyte quality. In contrast, antioxidant supplementation effectively alleviates MP-mediated oxidative injury (115).

#### 4.2.7 Gut microbiota dysbiosis

Gut microbiota (GM) plays a vital role in maintaining host metabolic homeostasis and immune function (116). GMs synthesize a variety of metabolites that enter ovarian tissues via the systemic circulation and regulate ovarian hormone secretion and cellular function. MPs damage intestinal structure and tight junctions, increase intestinal permeability, and alter GM composition. A compromised intestinal barrier allows microbiota, endotoxins, and MP particles to enter the circulation, further inducing oxidative stress and inflammation in the ovaries. Studies have confirmed that exposure to PS-MP significantly alters the composition of the GM in mice. Correlation analysis further revealed that the increased abundance of Bacteroidetes is positively associated with reproductive impairment, attributed to intestinal Th17-driven IL-17A inflammatory signaling (117). Combined exposure to MPs and phthalic acid esters induces severe intestinal inflammation and metabolic disorders in mice (118). MP contamination also leads to intestinal wall damage and microbial disturbance in zebrafish (119).

#### 4.2.8 Mechanism network of MP-induced reproductive toxicity

These mechanisms do not operate in isolation but rather form an interconnected pathological network that leads to dysfunction of the HPOA, ultimately resulting in infertility. Exposure to MPs can trigger GM dysbiosis and compromise the integrity of the intestinal barrier, facilitating the translocation of bacterial endotoxins and MPs into the circulatory system and leading to a chronic, low-grade systemic inflammatory state. This systemic inflammatory environment synergizes with MP-induced ovarian oxidative stress to activate pro-inflammatory signaling cascades, further exacerbating local ovarian inflammation and promoting the recruitment of immune cells to the follicular microenvironment. At the same time, MP-mediated epigenetic modifications, combined with hormonal imbalances, disrupt the transcriptional programs essential for steroidogenesis and folliculogenesis. These multiple factors are interrelated and act synergistically, ultimately inhibiting the secretion of gonadotropin-releasing hormone and gonadotropins, reducing estrogen and progesterone levels, and diminishing the ovaries’ responsiveness to gonadotropin stimulation. The ultimate consequences are arrested follicular development and atresia, accelerated ovarian fibrosis, and progressive depletion of ovarian reserve.

#### 4.2.9 Intergenerational transmission of toxic effects

MPs can exert intergenerational transmission effects. Emerging evidence has confirmed epigenetic alterations in zebrafish embryos following PS−MPs. Modified methylation patterns at stress−responsive gene loci, coupled with aberrant histone modifications, contribute to chromatin remodeling and disrupt gene expression during critical developmental windows (120). PS-NPs induce oxidative stress and DNA damage in human placental cells in a size- and charge-dependent manner (121). They activate the mitochondrial Bcl−2/caspase signaling pathway and autophagy, increase trophoblast apoptosis, and raise the risk of spontaneous abortion (122, 123). Exposure to MP in lactating mothers can be transmitted to offspring via breast milk and cause developmental abnormalities in juvenile animals (124). Furthermore, MPs may also impair lactation itself by inducing neuroinflammation in the hypothalamus, potentially suppressing the secretion of oxytocin and prolactin, which are critical for milk production and ejection. Combined exposure to MPs and other nanoparticles also produces synergistic reproductive toxicity (125).

## 5 Intervention measures

For infertile populations, particularly patients with unexplained infertility, the detrimental impacts of MPs on reproductive health cannot be overlooked. Early intervention can help mitigate the potential reproductive hazards posed by MPs. The application of antioxidants and anti−inflammatory agents can suppress inflammatory responses, alleviate oxidative stress, and thereby protect reproductive function. Relevant studies have demonstrated that probiotic supplementation inhibits the GM−driven IL−17A signaling pathway activated by PS−MPs, ultimately relieving excessive inflammation (126). Adopting a healthy lifestyle, including a balanced diet, moderate physical activity, and avoidance of hazardous substance exposure, can enhance the reproductive system’s antioxidant defense capacity and attenuate the adverse outcomes of MP exposure. Sun et al. collected 20 human endometrial specimens and successfully detected MPs in all samples via LDIR spectroscopy. Further analysis indicated that frequent consumption of milk tea, carbonated beverages, and chewing gum significantly elevated the abundance of multiple MP subtypes in the endometrium (127). Accordingly, prioritizing natural and organic diets and minimizing the intake of highly processed and packaged foods are effective strategies to reduce dietary MP ingestion.

In a targeted assessment focusing on seafood, salt, drinking water, and beer, drinking water contributed to more than 88% of total human MP intake within the studied dietary scope (128). Consuming filtered water and avoiding plastic-bottled water are strongly recommended. A variety of high−efficiency water purification devices are commercially available, and products with reliable MP removal performance should be preferentially selected. Washing reduces MP contamination in both raw and instant rice. However, even after washing, instant rice contains approximately three times more MPs than washed raw rice, suggesting that industrial processing introduces contamination that cannot be removed by simple washing (129). Daily plastic use ought to be reduced. Food containers are suggested to be replaced with food-grade glass and stainless steel; if ceramic utensils are used, those certified to be free of lead and cadmium are required. Regular household cleaning can improve indoor air quality by reducing the accumulation of indoor dust and synthetic fibers. The use of high-efficiency air purifiers also helps reduce exposure to MP in the respiratory tract. Furthermore, strengthening public environmental awareness, participating in environmental protection initiatives, and supporting policies to control plastic pollution can substantially reduce plastic waste emissions at the source.

## 6 Conclusion

Infertility has evolved into a severe global public health challenge. Exposure to MP induces endocrine and metabolic disorders, GM dysbiosis, inflammatory cascades, and oxidative stress in the human body. These pathological changes further impair tissue structural integrity, disrupt substance metabolism, and dysregulate the expression of key functional genes, thereby ultimately exerting toxic damage to the female reproductive system. Studies addressing MP-induced reproductive toxicity are still at an early phase and mainly concentrate on phenotypic changes. Future investigations should address the following limitation (1). Restricted by ethical constraints, technical bottlenecks, and safety concerns, direct clinical studies regarding the reproductive hazards of MPs remain scarce, and large−scale human epidemiological evidence is still lacking. Long−term biological effects of chronic human exposure to MP, as well as population−specific distribution characteristics and health impacts, require in−depth exploration (2). The migration pathways by which MPs enter multiple exposure routes, including the digestive, skin, and respiratory tracts, and subsequently target the ovary and uterus, remain poorly elucidated (3). A notable discrepancy exists between environmentally realistic MP exposure and laboratory experimental conditions. The exposure doses used in current animal and *in vitro* studies are generally far higher than those encountered in real environmental settings (4). Most existing laboratory studies adopt a single type of MP as the experimental pollutant. However, environmental MP pollution presents as complex mixtures with diverse components. Evidence concerning combined toxicity and the corresponding health risks of co−existing contaminants remains insufficient. Researchers should address the aforementioned limitations and fully elucidate how certain lifestyle modifications can harm women’s reproductive health. Although exposure to MP is ubiquitous, optimized public health strategies can significantly reduce the associated health risks. Therefore, recognizing MPs as an emerging risk factor for infertility and actively implementing targeted preventive strategies is of great significance for public health maintenance and clinical reproductive practice.
